# Resection of Clavicle

**Published:** 1864-10

**Authors:** R. L. Johnson

**Affiliations:** Assistant Surgeon P. A. C. S.


Art. III.—*Resection of Clavicle.* Reported by R. L. Johnson, Assistant Surgeon P. A. C. S.

Corporal Wm. H. Husky, Co. "I," 3d S. C. Regiment; wounded on the 18th November 1863; left hand amputated about an inch above the wrist November 19th; one-half of right clavicle resected December 27th, 1863.

*History.*—While in the act of shooting, a ball (Enfield) struck his *left* hand, injuring it so severely that it was amputated on the 19th; and, after passing through many folds of blanket, struck the *right* clavicle about two inches from the sternal articulation, fracturing and comminuting the bone for the space of about two inches, and splitting the sternal end to within half an inch of the articulation. It then passed, as I afterwards learned from a post mortem examination through the upper part of the humerus, entering at and breaking the inferior edges of the cartilaginous surface of the head of the bone, and coming out near the upper part of the attachment of the teres major and latissimus dorsi.

The case came under my charge on the 4th of December, at which time nothing was known of the position of the ball The surgeon who first examined the case was necessarily ignorant of the fact that the humerus was injured, for it was not broken in two but shot through, which caused, however, some splintering. December 5th.—Wrist (stump) doing well; right arm very much swollen, probably on account of an abscess in the anterior part of the shoulder, which presses on the veins. December 16.—Ligatures came away from the stump. December 19th.—Ordered whiskey, eggs, milk and other nourishing diet, which have just been obtained for the first time. December 24th.—The discharge of pus is now from one to two gills per day. It escapes by overflowing of the wound at the clavicle, the quantity being much augmented by pressure on the anterior part of the shoulder, over the abscess. December 25th.—Dr. Spinks, of Humphrey's brigade, and Dr. North, of Anderson's, were consulted to-day, and it was agreed, 1st. To put the patient under the influence of chloroform, in order to make a thorough examination. 2d. To remove the ball if its position could be ascertained. 3d. If the shoulder-joint has not been injured, or any other serious injury been done by the ball in its progress, to remove all necrosed portions of the clavicle. December 26th.—Through the kindness of one of the Federal surgeons at Knoxville, I procured a good and complete set of resecting instruments. December 27th.—The following operation was made to-day, Assistant-Surgeons Spinks, Pygott and Cotten and Dr. Allen being present: The patient having been anæsthetized, was placed upon the table, and a large gum catheter was introduced into the wound and passed outwards along the sinus to the anterior part of the shoulder, where there was a collection of pus, and where the ball was supposed to be. Nothing more was gained by this step than a knowledge of the exact position of the abscess.

The arm was then moved about to ascertain whether or not the humerus was fractured; and as there was no crepitus, no displacement and no impairment of the movements of the joint, it was decided that that the humerus and shoulder-joint were intact. An incision about two inches long was then made at the anterior edge of the deltoid muscle, and parallel with it, reaching from opposite the head of the humerus to below the neck After cutting nearly an inch deep through the swollen tissues, the knife entered the abscess. The finger was then introduced, and a large abscess was found with one sinus leading to the wound at the clavicle, and one leading around under the skin and fascia to another abscess which lay in the posterior part of the shoulder. The first or anterior abscess contained pus and a few small spiculæ of bone. The second or posterior contained dark, filthy pus and the ball which was extracted.

The clavicle was then resected.

The existing orifice was enlarged by incisions—one extending nearly to the articulation of the left clavicle and sternum, the other extending over the distal fragment for about two inches. The sternal end was then disarticulated and removed by dissection. All spiculæ were then carefully removed from the wound. These spiculæ were generally furnished on one surface with periosteum, by which they grew to the tissues. Their other surfaces being free, and acting as foreign bodies, were surrounded by pus. There was one point, however, at the bottom of the wound, about one inch and a half long, which was firm and immovable and covered with healthy granulations. This was supposed (at the time) to be a portion of the clavicle from the posterior surface that had never been displaced, and as there was no collection of pus under or around it, it was not removed.

A chain saw was then passed under the distal fragment an inch from the broken end, the bone sawn in two, and the fragment removed by dissection. Though no veins or arteries of any size were cut, he lost over half a pint of blood, the tissues being very vascular. A few sutures were taken; wet lint was applied; the patient was put to bed and morphine and whiskey were administered. December 28th.—Rested well last night; very pale and languid to-day; without appetite and with some diarrhœa; a counter opening was made into the abscess from which the ball had been extracted; prescribed one grain of opium, ordered eggs, whiskey, &c. December 29th.—Has a little more color in his cheeks today than he had yesterday; eat squirrel stew with great relish; bowels better; prescribed tr. catechu, gave whiskey, eggs, milk, &c.; the openings in the shoulder are discharging freely to-day, the discharge from the wound being much diminished. The swelling of the arm is so much reduced as to slacken the bandages, the first of which were applied on the 5th inst., and which had been re-applied every two or three days since. The arm reduced about one-third. December 30th and 31st.—Doing well. January 1st, 1864.—Doing well; appetite good; wound and incisions granulating; discharge from the wound much diminished, there not being enough to overflow the wound in twelve hours; the discharge from the anterior incision (in which a tent is kept) which is now the outlet from the abscess in the shoulder is less than the discharge was from the wound before the operation. January 7th.—Healthy granulations over the end of the bone, and, indeed, everywhere about the wound; discharge, which is from the shoulder, very slight. January 7th.—In the afternoon, some diarrhœa; prescribed tr. opii, tr. catechu in equal parts, twenty drops after each operation on bowels. January 14th.—Whiskey supplies have been out since the 7th; diarrhœa has been *constant;* prescribed tannin, catechu, opium, &c., with no effect; losing flesh and getting weaker. January 21st.—Diarrhœa constant; patient very weak; procured whiskey to-day. January 26th.—During past six days have had good supply of stimulants; patient very weak; diarrhœa continues; will probably not live till to-morrow. January 27th.—Died at one o'clock, P. M.

*Post Mortem.*—On making an incision from the wound, which had healed to a considerable extent, to the incision on on the front part of the shoulder, and from there around to the posterior incision I found the track of a large abscess. This also extended downwards in front, and parallel with the pectoralis minor. At the bottom of this branch abscess there was a spicula of bone half an inch long. The ball had passed through to the head of the humerus, but had not broken it in two. *It did not* pass therefore in *front* of the *shoulder*, through the sinus by which it was extracted. Wherever any periosteum was left, bone was forming rapidly. The hard, firm place in the bottom of the wound, supposed during the operation to be a spicula from the posterior part of the clavicle, proved to be entirely new bony formation. The abscesses around the head of the humerus were large, and had burrowed back into the shoulder to some extent.

